# Effect of cell salvage on bleeding, transfusion of blood products, and bleeding parameters in patients undergoing liver transplantation with intraoperative massive blood transfusion

**DOI:** 10.55730/1300-0144.5437

**Published:** 2022-08-05

**Authors:** Tümay ULUDAĞ YANARAL, Pelin KARAASLAN

**Affiliations:** Department of Anesthesiology and Reanimation, Faculty of Medicine, İstanbul Medipol University, İstanbul, Turkey

**Keywords:** Liver transplantation, bleeding, autologous blood transfusion, red blood cell transfusion

## Abstract

**Background/aim:**

Autologous transfusion using a cell saver system has been used in liver transplantation with controversial outcomes. Its efficiency in patients with massive intraoperative transfusion has not been studied yet. This study aimed to evaluate effect of cell salvage (CS) on intraoperative bleeding and transfusion practices in liver transplantation with massive intraoperative transfusion.

**Materials and methods:**

Consecutive patients aged ≥18 years with intraoperative massive blood transfusion (≥ 6 units) between March 2014 and September 2020 were included. Patients subjected to CS were grouped as CS, whereas other patients were grouped as control. Number of transfused red blood cells was study’s primary outcome.

**Results:**

There were 38 and 32 patients in CS and control groups, respectively. Median blood loss was significantly lower in CS group than in control group (2500 mL vs. 4000 mL, p = 0.010). There were significantly more transfusions of red blood cells, fresh frozen plasma, platelets, and cryoprecipitates in CS group (p < 0.05). Postoperative median hemoglobin levels were determined as 4.8 g/dL and 8.2 g/dL in CS and control groups (p < 0.001). The decrease in postoperative hemoglobin levels compared to preoperative values was significantly higher in the CS group (p < 0.001). The mortality rate in postoperative first year was significantly higher in CS group than in control group (36.8% vs. 12.5%, p = 0.041).

**Conclusion:**

Use of CS in patients undergoing liver transplantation with massive intraoperative transfusion did not improve clinical and transfusion-related outcomes. In conclusion, its usage can be questionable given the absence of any clinical benefit and presence of poor outcomes.

## 1. Introduction

Liver transplantation is a unique treatment modality indicated for different end-stage liver diseases [1]. It has a variable intraoperative bleeding potential [2,3]. Coagulopathy due to the underlying chronic liver diseases and surgical complexity are the major risk factors for massive bleeding in these patients [2]. The intraoperative blood loss and blood product requirements during liver transplantation have considerably decreased during the course of the last several decades; yet large volume transfusion of red blood cells (RBC) and blood products are still required for the management of massive intraoperative bleeding in 10% to 20% of the cases [3–5].

The patient blood management strategy includes allogeneic blood transfusions and autologous cell salvage techniques [2,4]. Each modality has its advantages and disadvantages as per the outcomes of liver transplantation. Massive transfusion has been found to be associated with decreased patient and graft survival rates [1,6] and with an increased risk of transfusion-related complications such as profound coagulopathy, acute lung injury, and acute kidney injury [5]. The limited blood products and exposure to viral, bacterial, and protozoal diseases are the primary concerns disfavoring allogeneic blood transfusions [1]. Autologous transfusion using a cell saver system is another technique used to manage bleeding during major surgical procedures such as liver transplantation, coronary artery bypass grafting, and other types of operations with significant bleeding risk [1,7–10]. Some reports revealed that higher blood losses might be seen during liver transplantation due to fibrinolysis developed in association with cell salvage (CS) [11,12]. The cost of the technique is another primary concern preventing its widespread use [1,3,13]. Although its use has been associated with reducing blood transfusion, its impact on the early and long-term outcomes is unclear [1,3,5,7,14]. Despite the data available on the use of CS in liver transplantation in the literature, to date, there is no study in which the benefit and reliability of CS were evaluated in patients requiring massive blood transfusion. Additionally, the relationship between the coagulative and fibrinolytic laboratory parameters and CS has also not been studied in detail.

In view of the foregoing, the objective of this study is to evaluate the effect of the administration of autologous transfusion using a cell saver system on the amount of intraoperative bleeding, transfusion practices, laboratory investigations for bleeding disorders, and on the outcomes in liver transplantation patients with massive intraoperative transfusion.

## 2. Materials and method

### 2.1. Study design

The patients who underwent liver transplantation between March 2014 and September 2020 in İstanbul Medipol University Hospital were retrospectively evaluated. The institutional ethics committee approved the study (approval number: 2020/975). The study was carried out in accordance with the principles of the Declaration of Helsinki.

### 2.2. Population and sample

The population of the study comprised all consecutive patients who required massive intraoperative transfusion (≥6 transfusions of RBC units) [15]. The exclusion criteria were determined as having an age <18 years, incomplete data, deceased donor transplantation and congenital bleeding disorders. The patients who met the study inclusion criteria were divided into two groups; the group of patients with intraoperative CS, that is, the CS group, and the group of patients without CS, that is, the control group (Figure).

### 2.3. Anesthesia protocol and surgical technique

The monitoring and anesthesia protocol used in this study was previously described in the literature [13–16]. For the surgical approach, hepatectomy preserving the recipient’s inferior vena cava was undertaken using the piggy-back technique (the classic technique).

#### Blood analysis

Serial arterial blood gas analysis included hemoglobin, potassium, and ionized calcium measurements. Intraoperative measurements of INR (international normalized ratio) and platelet count were used to monitor the coagulation profile of the patients. The decision to carry out a transfusion during the surgery was made jointly by the surgeon and the anesthesiologist based on the patient’s hemodynamic status, blood loss, and hemoglobin concentration [7]. Accordingly, in cases where deemed necessary, the Rapid Infuser System (Belmont Instrument Corp., Billerica, MA) was used to administer the blood product.

Crystalloid and colloid solutions were used intravenously to make up for the volume loss. If a patient received a transfusion, the tests with abnormal results were repeated and dealt with in accordance with the algorithm while the patient was in the operating room and later on in the intensive care unit.

In the event that the patient’s hemoglobin (Hb) level was found to be below 8 g/dL, an allogeneic red blood cell (RBC) transfusion was performed. In the CS group, allogeneic RBC was performed if the Hb level did not rise above 8 g/dL despite the retransfusion of all autologous blood. For Hb levels of 8 to 10 mg/L, the transfusion decision was made based on symptoms and signs of anemia. If bleeding persisted, the allogeneic fresh frozen plasma (FFP), platelet, and antifibrinolytic agent (tranexamic acid) transfusion were used in accordance with the thromboelastography guidance [14]. A kaolin-activated thromboelastography assay was performed using a 5000 series analyzer (Haemoscope, Inc, Niles, Illinois).

### 2.4. Cell saver technique

The cell saver system was not used in liver transplant patients with malignancy [1]. The Cell Saver (Haemonetics, Braintree, MA, USA) machine was prepared with a two-suction system before the surgery. As the standard protocol, heparinized saline solution with 5000 IU of heparin in 1 L of 0.9% saline solution was used at a rate of 100 mL/h to prevent thrombogenesis during blood collection. If the measured intraoperative hemoglobin level was 8 g/dL or lower, the processed (filtrated, centrifuged, washed, and concentrated) blood was retransfused. The blood may contain about 0.002% of the prewash heparin.

### 2.5. Variables

The research data were collected from the hospital information system and the patients’ medical records. The patients’ demographic [age, gender, height, weight, body mass index (BMI)], clinical (smoking history, comorbidities, the Child-Turcotte-Pugh category, the Model for End-stage Liver Disease (MELD) score and subgroups, reasons for transplantation), laboratory, operative, and follow-up data were recorded.

The Child-Turcotte-Pugh scores were calculated for all patients and categorized from A to C [15]. The MELD score was calculated using the immediate preoperative values for INR, serum creatinine, total bilirubin, and primary etiology of liver failure [16,17].

In the control group, intraoperative blood loss was calculated by the addition of the blood volume in the suction container to the volume of blood collected with abdominal sponges and compresses. On the other hand, in the CS group, intraoperative blood loss was calculated by the addition of the blood volume in the cell saver system to the blood loss calculated for the control group. The total intraoperatively transfused RBC, FFP, platelet, cryoprecipitate, and albumin values were recorded. The allogeneic RBC volume was 300 mL per unit. Each 300 mL of autologous blood retransfused in the CS group was recorded as 1 unit.

### 2.6. Follow-up

Postoperatively, patients were followed in the intensive care unit and extubated based on the results of the blood gas analysis, laboratory tests, and Doppler sonography. The patients were kept in the intensive care unit until they were hemodynamically stable and had good graft function. They were discharged from the hospital after it was established that they were clinically well.

The overall mortality rate was determined at the end of the postoperative third month and the first year. The follow-up data available in the hospital medical records were analyzed. Patients whose follow-up data were missing were given a call-in order to determine the overall mortality rates.

### 2.7. Statistical analysis

The number of transfused RBC and blood product units was the study’s primary outcome. The secondary outcomes were the changes in the laboratory results associated with the bleeding disorders and the mortality rates at the postoperative third month and the first year.

Descriptive statistics were expressed as mean ± standard deviation or median and minimum-maximum values in the case of continuous variables depending on the distribution pattern of the respective statistic. Categorical variables were expressed as numbers and percentage values. The conformity of the numerical variables with normal distribution was analyzed using the Shapiro-Wilk, Kolmogorov-Smirnov, and Anderson-Darling tests. The Independent Samples t-test was used to compare two independent groups in the case of numerical variables, which were determined to conform to normal distribution. The Mann-Whitney U test was used to compare two independent groups in the case of variables that were determined not to conform to normal distribution. Pearson’s chi-squared and Fisher’s exact tests were used to compare the differences between categorical variables in 2 × 2 tables. The Fisher-Freeman-Halton test was used in RxC tables. The Wilcoxon signed-rank test was used to compare the differences of the laboratory parameters in two different intervals.

For statistical analysis, Jamovi project (2021) (Jamovi version 2.2.2.0) [computer software, retrieved from https://www.jamovi.org) and JASP 0.16 (JASP version 0.16; retrieved from https://jasp-stats.org) software packages were used. In all statistical analyses, the significance level (p-value) was set at 0.05.

## 3. Results

Seventy patients with massive intraoperative transfusion who met the study inclusion criteria were included in the study. CS was used in 38 (54.3%) of these patients. The groups were similar in the demographic and clinical characteristics except for the incidence of hypertension (p = 0.033) and the distribution of the preoperative diagnoses (p = 0.017). A significantly higher number of patients in the CS group had the transplantation due to HCV (Hepatitis C virus) and cryptogenic causes. The median value of the MELD score was similar in both groups (p = 0.140). The demographic and clinical characteristics of the patients are given in Table 1.

There were significant differences between the CS and control groups in terms of preoperative laboratory test results (Table 2). The median hemoglobin level was significantly higher in the CS group than in the control group (10.9 g/dL vs. 8.7 g/dL, p < 0.001). In the CS group, the prothrombin time and aPTT (activated partial thromboplastin time) were significantly lower than those of the control group (p = 0.020 and p = 0.023; respectively). Other preoperative measurements were similar between the groups (p > 0.05).

The intra- and intergroup comparisons of the postoperative laboratory investigations are detailed in Table 2. There were significant differences in the laboratory measurements between the preoperative and postoperative values within each group and between the postoperative values of the two groups. The postoperative median hemoglobin levels were 8.2 g/dL and 4.8 g/dL in the control and CS groups, respectively (p < 0.001). The postoperative prothrombin time was significantly lower in the CS group than in the control group (22 s vs. 28.4 s; p = 0.001). The changes between the preoperative and postoperative laboratory values within each group are given in Table 2.

The perioperative characteristics of the study groups are shown in Table 3. There was no difference between the groups in duration of the surgery, anhepatic phase, and warm ischemia; however, the cold ischemia time was significantly lower in Group CS (p = 0.001). A significantly higher number of patients in the CS group required a high-dose vasopressor (p = 0.010). The median lactate level was significantly higher in the CS group than in the control group (8.4 vs. 4.4 mmol/L, p < 0.001). There was no significant difference between the groups in the total volume of the infused intravenous fluid (p = 0.073). However, the crystalloid fluid volume used in the CS group was significantly lower than that of the control group (6000 mL vs. 7250 mL, p = 0.034). The median blood loss was 4000 mL in the control group. Additionally, the intraoperative blood loss was significantly lower in the CS group than in the control group (2500 mL, p = 0.010).

The median values of the transfused allogeneic RBC were eight and three units in the control and CS groups, respectively, and significantly higher in the control group (p < 0.001). The median amount of total RBC, including the units salvaged for autologous transfusion and the transfused allogeneic RBC, was 12.6 units. The transfused total RBC and FFP were significantly higher in Group CS than in the control group (p < 0.001 in both cases) (Table 4). The distribution of other transfused blood products is shown in Table 4.

The percentage changes between the postoperative and preoperative laboratory values within each group are given in Table 5. There was a significant difference between the groups in the percentage change in hemoglobin levels (p < 0.001). The decrease observed in the postoperative hemoglobin levels compared to the preoperative values was significantly higher in the CS group than in the control group.

The length of hospital stay was significantly lower in the CS group (p = 0.021). The mortality rate in the third-month follow-up was comparable between the groups (p = 0.314), yet there were more mortalities in the CS group than in the control group in the first year (36.8% vs. 12.5%, p = 0.041) (Table 6).

## 4. Discussion

The findings of this study did not support the use of CS during liver transplantation since there was no improvement in the clinical outcomes and the laboratory parameters related to bleeding disorders. Although the amount of allogeneic blood transfusion was significantly decreased, there was a higher number of transfused blood products, including both the allogeneic and salvaged blood, in the CS group. In contradiction with the significantly lower blood loss in the CS group, the total transfused RBC units were significantly higher, and the postoperative hemoglobin levels were significantly lower in the CS group. Based on the study findings, CS usage was deemed questionable given the absence of any clinical benefit and the presence of possible adverse effects.

The amount of intraoperative blood loss in liver transplantation has been regarded as a prognostic factor affecting the survival and retransplantation rates via unknown mechanisms [2,4]. A cut-off value of >6 units or >1100 mL blood transfusion has been associated with the worse short-term and long-term outcomes [5,15,18,19]. These problematic issues have raised questions about the indications and amount of blood and blood product transfusions. Different cut-off values were used in the previous studies for the number of transfused blood products and the intervals to define massive blood transfusion in liver transplantation. The transfusion of at least four [20], six [15,16,21] or ten units [5,22–25] of RBC in the intraoperative period [15,20,22–24] or up to the postoperative 24 h [16,21,25] to 48 h [5] were regarded as the criteria as per this definition. The transfusion of 6 or more RBC units was accepted as the cut-off value for the definition of massive intraoperative transfusion [15]. The differences in the amount and the interval may lead to controversial outcomes.

In order to avoid unnecessary allogeneic blood transfusions and minimize the amounts thereof during major surgical procedures, reinfusion of blood collected in the surgical field has been developed since 1885 [2]. The reduction in the need for allogeneic transfusions is directly related to a decrease in the cost and the rate of adverse effects of the transfusion. It has also been speculated that the rate of surgical infections, the length of hospital stay, and the treatment-related costs would decrease [26]. Hence, there is a need to determine the indications and benefits of the CS approaches in liver transplantation [25].

The use of the CS approaches has been analyzed in previous studies. Similar to the results of other studies, a reduction in allogeneic heterologous blood transfusions was observed in this study in liver transplants [1,3,13,19]. Kırnap et al. demonstrated considerable reductions in the allogeneic blood transfusions during liver transplantation [1]. They reported that CS decreased the need for blood transfusions from 20–25 mL/kg to 5–10 mL/kg yet with no changes in the transfusion rate, including both the allogeneic and salvaged transfusions [1]. In Massicotte’s study, the mean number of transfused allogeneic RBC units was found as 0.4 per patient regardless of the use of the CS [3]. Substantial reductions in fresh frozen plasma and platelet transfusions were reported with the use of CS in other studies, contrary to the findings of this study [13,19]. In this study, the use of CS was associated with a higher number of transfused blood and other blood products. The median transfused RBC units were determined as 8 and 12.6 in the control and CS groups, respectively. The fact that only patients with massive intraoperative transfusion ( ≥6 units) were included in this study was the most significant difference of this study that separated it from other studies [1,3,13,19].

Due to the lack of clotting factors and platelet on the washed RBC, an increased blood loss and the requirement for blood products may occur [25]. The increased rates of acute kidney injury secondary to the increased hemolysis and the salvaged blood syndrome and the cost of the CS system are the other disadvantages. Massicotte et al. reported an increased blood loss with the use of CS, which they attributed to two new junior surgeons and speculated that two RBC unit transfusions could be saved using CS [3]. Hendriks et al. reported a significantly higher number of RBC transfusions in patients who underwent liver transplantation with the use of CS [11]. They concluded that the use of autologous blood was a poor prognostic factor for the transfusion requirements. In earlier studies, more RBC, fresh frozen plasma, cryoprecipitate, and platelets transfusions were needed in patients who were administered autologous transfusion using CS [27,28], which was attributed to hyperfibrinolysis developed secondary to the release of fibrinolytic compounds from blood cells in the collected blood and the transplanted liver [11].

In comparison, in this study, more transfusions were performed in the CS group, albeit the blood loss was significantly lower. The rate of the intraoperative high-dose vasopressor treatment was also higher. The postoperative hemoglobin level was significantly lower, along with the use of more transfusions. Given the study’s retrospective nature, it has proven difficult to analyze the cause-and-effect relationships between the use of CS and higher requirement for RBC transfusions. The increased mortality rate in the postoperative first year needs to be explained in detail since the use of CS alone does not explain the relevant finding. Therefore, further prospective studies are needed to clarify the results of this study.

There were also some limitations to this study. The primary limitations were the retrospective design, small sample size, lack of match-pair analysis, absence of fibrin degradation products, absence of emergency and elective transplantation data. Additionally, there was difficulty in analyzing the reciprocal relations between the CS system and intraoperative bleeding. Lastly, there might have been a selection bias considering the differences in the preoperative hemoglobin levels between the groups.

In conclusion, the findings of this study revealed that the CS approach caused significant reductions in the number of allogeneic blood transfusions. Nevertheless, other transfusion-related parameters were poor in patients with CS. Therefore, the use of CS in patients undergoing liver transplantation with intraoperative massive blood transfusion should be reevaluated since it does not seem to improve the clinical outcomes or the transfusion practices. Prospective, randomized, large-scale studies are needed on groups with similar demographic data and longer follow-up periods.

## Figures and Tables

**Figure:**
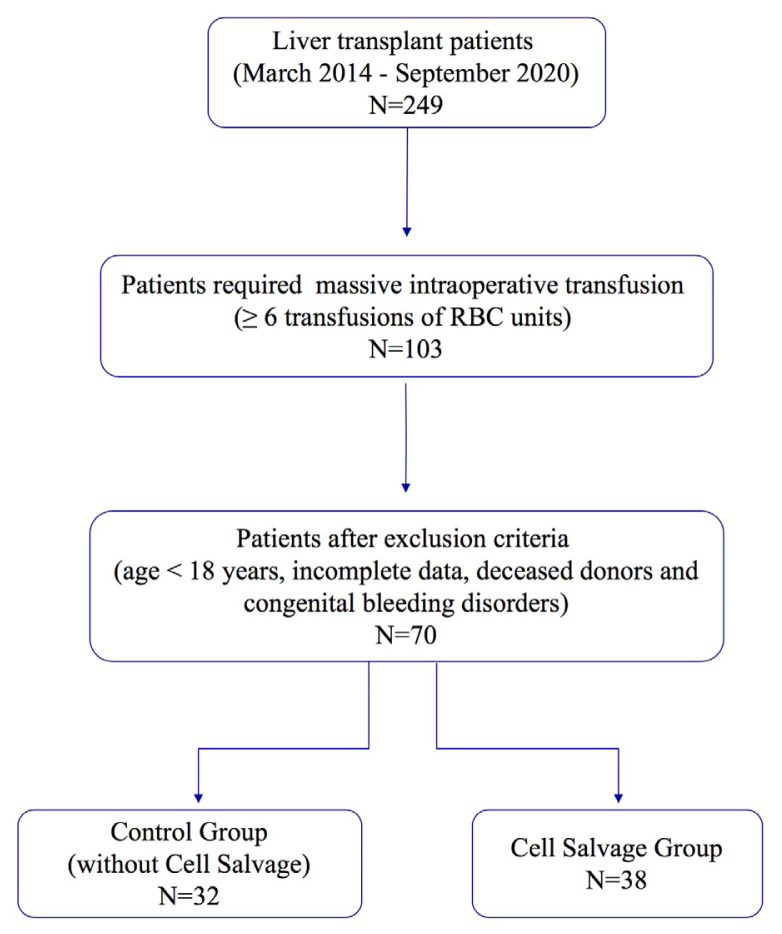
Flow chart of research methodology.

**Table 1:** Demographic and clinical characteristics of the study groups.

	Overall (n = 70)	Control group (n = 32)	Group CS (n = 38)	*p*
**Age (year)** †,‡	53.7 ± 11.1	54.1 ± 11.7	53.3 ± 10.6	0.658***
	55.0 [24.0–72.0]	55.5 [28.0–72.0]	55.0 [24.0–71.0]	
**Sex** §				0.773*
Male	48 (68.6)	23 (71.9)	25 (65.8)	
Female	22 (31.4)	9 (28.1)	13 (34.2)	
**Height (cm)** †	165.3 ± 9.4	165.8 ± 9.5	164.8 ± 9.4	0.691**
**Weight (kg)** †	75.2 ± 14.5	72.3 ± 13.7	77.5 ± 14.9	0.135**
**Body mass index (kg/m^2^)** †	27.5 ± 5.3	26.5 ± 5.1	28.3 ± 5.5	0.169**
**Smoking history** §	25 (36.2)	11 (34.4)	14 (37.8)	0.962*
**Comorbidities** §	41 (58.6)	21 (65.6)	20 (52.6)	0.392*
**Type of comorbidity** §				
Diabetes mellitus	30 (42.9)	14 (43.8)	16 (42.1)	0.999*
Hypertension	15 (21.4)	11 (34.4)	4 (10.5)	**0.033** *
Coronary artery disease	16 (22.9)	10 (31.2)	6 (15.8)	0.212*
Chronic renal failure	5 (7.1)	3 (9.4)	2 (5.3)	0.654*
**Child score** §				0.058*
A	4 (5.7)	2 (6.2)	2 (5.3)	
B	32 (45.7)	10 (31.2)	22 (57.9)	
C	34 (48.6)	20 (62.5)	14 (36.8)	
**MELD score** ‡	17.5 [8.0–40.0]	20.0 [11.0–27.0]	16.0 [8.0–40.0]	0.140***
**Reasons for transplantation** §				**0.017** *
Hepatitis B	9 (12.9)	3 (9.4)	6 (15.8)	
Hepatitis B + hepatitis D	4 (5.7)	2 (6.2)	2 (5.3)	
Hepatitis C	8 (11.4)	2 (6.2)	6 (15.8)	
Alcoholic cirrhosis	8 (11.4)	5 (15.6)	3 (7.9)	
Nonalcoholic steatohepatitis	7 (10.0) a	7 (21.9) b	0 (0.0)	
Cryptogenic	21 (30.0)	6 (18.8)	15 (39.5)	
Autoimmune	2 (2.9)	1 (3.1)	1 (2.6)	
Primary biliary sclerosis	2 (2.9)	0 (0.0)	2 (5.3)	
Others	9 (12.9)	6 (18.8)	3 (7.9)	

† mean ± standard deviation,

‡ median [min-max],

§ n (%)

CS: cell salvage, MELD: Model for end-stage liver disease.

* Pearson chi-square, Fisher’s exact or Fisher Freeman Halton tests

** Independent samples t-test

*** Mann-Whitney U test

**Table 2:** Comparison of the preoperative and postoperative laboratory investigations between the groups.

	Control group (n = 32)	Group CS (n = 38)	*p* *
**Hemoglobin (g/dL)** ‡			
Preoperative	8.7 [7.2–13.0]	10.9 [7.8–14.9]	**<0.001**
Postoperative	8.2 [6.9–10.0]	4.8 [1.5–13.3]	**<0.001**
**p** **	**0.027**	**<0.001**	
**Platelet count (x 10^3^/μL)** ‡			
Preoperative	73.5 [14.0–390.0]	79.5 [27.0–180.0]	0.728
Postoperative	48.0 [21.0–270.0]	52.5 [17.0–113.0]	0.841
**p** **	**<0.001**	**<0.001**	
**Prothrombin time (s)** ‡			
Preoperative	21.6 [14.2–41.2]	18.0 [12.6–38.0]	**0.020**
Postoperative	28.4 [18.7–46.9]	22.0 [16.8–40.0]	**0.001**
**p** **	**<0.001**	**0.002**	
**INR** ‡			
Preoperative	1.6 [1.1–3.1]	1.5 [1.0–5.6]	0.750
Postoperative	2.3 [1.5–3.5]	2.2 [1.3–3.4]	0.283
**p** **	**<0.001**	**<0.001**	
**APTT (s)** ‡			
Preoperative	40.0 [26.0–67.6]	37.0 [24.5–59.0]	**0.023**
Postoperative	42.0 [33.0–68.3]	39.6 [32.0–107.0]	0.313
**p** **	**0.039**	**0.002**	
**Fibrinogen (mg/dL)** ‡			
Preoperative	184.5 [101.0–580.0]	246.5 [84.0–468.0]	0.135
Postoperative	110.5 [64.0–201.0]	110.0 [50.0–250.0]	0.919
**p** **	**<0.001**	**<0.001**	
**Creatine (mg/dL)** ‡			
Preoperative	0.9 [0.3–2.9]	0.9 [0.4–4.3]	0.786
Postoperative	0.9 [0.3–2.4]	1.2 [0.5–3.4]	**0.002**
**p** **	0.252	**0.004**	
**GFR (mL/dk/1.73 m^2^)** ‡			
Preoperative	82.8 [23.0–222.4]	87.3 [14.1–264.3]	0.846
Postoperative	77.0 [27.0–222.4]	61.4 [17.6–124.2]	**0.006**
**p** **	0.094	**<0.001**	
**Total bilirubin (mg/dL)** ‡			
Preoperative	2.2 [0.2–21.4]	2.1 [0.4–33.0]	0.641
Postoperative	5.0 [2.2–15.0]	6.6 [2.1–20.0]	**0.021**
**p** **	0.308	**0.001**	
**Direct bilirubin (mg/dL)** *			
Preoperative	1.4 [0.1–19.1]	1.2 [0.1–29.0]	0.827
Postoperative	2.5 [0.7–10.5]	2.7 [0.4–11.0]	0.308
**p** **	0.911	0.087	
**AST (U/L)** ‡			
Preoperative	41.8 [14.8–373.0]	50.0 [23.0–2625.0]	**0.030**
Postoperative	144.8 [86.0–1137.0]	343.5 [109.0–2251.0]	**<0.001**
**p** **	**<0.001**	**<0.001**	
**ALT (U/L)** ‡			
Preoperative	21.5 [5.0–263.0]	30.5 [14.0–2612.0]	**0.044**
Postoperative	128.4 [70.0–959.0]	374.0 [53.0–1852.0]	**<0.001**
**p** **	**<0.001**	**<0.001**	
**Albumin (mg/dL)** ‡			
Preoperative	3.0 [2.0–4.3]	2.9 [2.2–5.4]	0.855
Postoperative	3.4 [2.8–4.3]	3.1 [1.5–3.8]	**0.001**
**p** **	**0.002**	0.612	

‡ median [min-max]

CS: cell salvage, INR: international normalized ratio, aPTT: activated partial thromboplastin time, GFR: glomerular filtration rate, ALT: alanine aminotransferase, AST: aspartate aminotransferase

* Mann-Whitney U test

** Wilcoxon test

**Table 3:** Perioperative characteristics of the study groups.

	Control group (n = 32)	Group CS (n = 38)	*p*
**Graft weight/recipient weight** ‡	1.3 [0.8–2.0]	1.2 [0.6–2.0]	0.829***
**Operative time (min)** ‡	540.0 [390.0– 807.0]	480.0 [360.0– 840.0]	0.151***
**Duration of anhepatic phase (min)** ‡	74.5 [38.0–370.0]	59.5 [35.0– 310.0]	0.184***
**Cold ischemia time (min)** ‡	36.0 [15.0–116.0]	22.0 [10.0– 55.0]	**0.001** ***
**Warm ischemia time (min)** ‡	47.5 [17.0–136.0]	41.0 [25.0– 107.0]	0.107***
**Need for vasopressors**	30 (93.8)	36 (94.7)	0.999*
Low dose	10 (33.3)	2 (5.6)	**0.010** *
High dose	20 (66.7)	34 (94.4)	
**CVP-preoperative (cmH_2_O)** †	11.4 ± 4.6	11.1 ± 2.9	0.762**
**CVP-postoperative (cmH_2_O)** †	7.7 ± 2.4	7.1 ± 3.1	0.327**
**Lactate (mmol/L) intraoperative** ‡	1.1 [0.4–2.9]	1.2 [0.6–2.0]	0.953***
**postoperative** ‡	4.4 [2.2–12.0]	8.4 [3.6–13.0]	**<0.001** ***
**Total intravenous fluid (mL)** ‡	12,360.0 [5810.0– 20,400.0]	14470.0 [7080.0–28,400.0]	0.073***
**Crystalloids (mL)** ‡	7250.0 [3000.0– 16,000.0]	6000.0 [3000.0 –20,000.0]	**0.034** ***
**Colloids (mL)** ‡	500.0 [500.0– 1100.0]	500.0 [500.0– 2000.0]	0.511***
**Ascites volume (mL)** ‡	2750.0 [1000.0– 9700.0]	2250.0 [1000.0 –10,000.0]	0.387***
**Total blood loss (mL)** ‡	4000.0 [1500.0– 8000.0]	2500.0 [1350.0 –18,000.0]	**0.010** ***
**Urine volume (mL)** ‡	2150.0 [450.0– 9000.0]	1800.0 [400.0– 4500.0]	0.051***

† mean ± standard deviation,

‡ median [min-max],

§ n (%)

CS: cell salvage, CVP: central venous pressure

* Pearson chi-square or Fisher’s exact test

** Independent samples t-test

*** Mann-Whitney U test

**Table 4:** Comparison of the intraoperative transfusion practices in the groups.

Number per patient	Control group (n = 32)	Group CS (n = 38)	*p*
**Allogeneic red blood cell (unit)** ‡	8.0 [6.0–12.0]	3.0 [0.0–21.0]	**<0.001** ***
**Total red blood cell (unit)** ‡	8.0 [6.0–12.0]	12.6 [6.1–72.0]	**<0.001** ***
**Fresh frozen plasma transfusion (unit)** ‡	5.0 [0.0–12.0]	13.0 [5.0–32.0]	**<0.001** ***
**Thrombocyte transfusion (unit)** ‡	0.0 [0.0–4.0]	1.0 [0.0–6.0]	**0.025** ***
**Cryoprecipitate transfusion (unit)** ‡	2.0 [0.0–12.0]	0.0 [0.0–6.0]	**0.004** ***
**Albumin (20%–100 mL) replacement** ‡	7.0 [3.0–15.0]	4.0 [0.0–6.0]	**<0.001** ***

‡ median [min-max]

CS: cell salvage

* Pearson chi-square or Fisher’s exact test

** Independent samples t-test

*** Mann-Whitney U test

**Table 5:** Percent changes between the postoperative and preoperative laboratory parameters in the study groups.

Δ %	Control group (n = 32)	Group CS (n = 38)	*p*
**Hemoglobin** ‡	−7.8 [−30.4−29.9]	−55.0 [−81.2−50.0]	**<0.001**
**Platelet count** ‡	−28.9 [−83.7−192.9]	−35.0 [−78.3−62.3]	0.383
**Prothrombin time** ‡	34.8 [−28.2−208.6]	22.0 [−42.9−100.0]	0.294
**INR** ‡	40.8 [−29.0−219.8]	41.5 [−69.6−130.6]	0.976
**APTT** ‡	9.0 [−39.9−71.2]	8.9 [−20.0−178.4]	0.349
**Fibrinogen** ‡	−43.0 [−88.3−14.3]	−54.6 [−74.5−31.0]	0.194

‡ median [min-max]

CS: cell salvage, INR: international normalized ratio, aPTT: activated partial thromboplastin time.

* Mann-Whitney U test

**Table 6:** Postoperative outcomes of the study groups.

	Control group (n = 32)	Group CS (n = 38)	*p*
**Length of hospital stay (day)** ‡	18.5 [7.0–90.0]	14.0 [1.0–58.0]	**0.021** ***
**Length of intensive care unit (day)** ‡	3.0 [1.0–20.0]	2.5 [1.0–21.0]	0.369***
**3rd month follow-up** §			0.314*
Survived	29 (90.6)	30 (78.9)	
Nonsurvived	3 (9.4)	8 (21.1)	
**12th month follow-up** §			**0.041** *
Survived	28 (87.5)	24 (63.2)	
Nonsurvived	4 (12.5)	14 (36.8)	

‡ median [min-max],

§ n (%)

CS: cell salvage

* Pearson chi-square, Fisher’s exact or Fisher Freeman Halton tests

** Independent samples t-test

*** Mann-Whitney U test
